# Theranostics in Developing Countries: Addressing Challenges and Potentials from Training to Practice

**DOI:** 10.1055/s-0043-1774733

**Published:** 2023-09-20

**Authors:** Akram Al-Ibraheem

**Affiliations:** 1 Department of Nuclear Medicine, King Hussein Cancer Center (KHCC), Amman, Jordan; 2 Division of Nuclear Medicine, Department of Radiology and Nuclear Medicine, University of Jordan, Amman, Jordan; 3 Arab Society of Nuclear Medicine (ARSNM), Amman, Jordan

In recent times, nuclear medicine (NM) has witnessed noteworthy expansion, presenting several technologies and procedures that aid in the identification and management of various illnesses and disorders. The current era of globalization has posed difficulties for health care systems in managing the escalating incidence of noncommunicable diseases (NCDs) in developing countries. 1 The use of radionuclide pairs for both diagnosis and treatment, known as theranostics, has become increasingly popular in developing countries. 2 This is due to the availability of more resources, expertise, and personnel. Although the idea of combining therapy and diagnosis has been studied and utilized for many years, recent advancements in cancer genomics and hallmarks have led to significant progress in the field of theranostics over the past two decades. Nowadays, NM theranostics are improving the prognosis of cancer patients through the use of ^177^ lutetium-based radionuclide therapies, which have been validated in phase III clinical trials with high confidence. 3 4

To date, many Southeast Asian, Middle Eastern, and Latin American countries have continuously offered advanced NM services. 5 6 7 Some of these countries serve as exemplary models in the developing world, as they leverage their limited resources to generate numerous radionuclides, conduct practical research projects, and significantly contribute to the progress of theranostics. Additionally, they have augmented the size of their NM workforce by offering specialized NM training programs as well as fellowship programs. Although theranostics has made substantial strides and improvements, a discernible discrepancy persists among developing countries. This disparity is particularly noticeable in the Middle East, where numerous countries are embroiled in wars and conflicts. 8 These developing countries are currently encountering difficulties keeping up with the latest advancements in theranostics. Presently, there are over 2,000 NM centers in the region. 9 Several countries in the region have made investments in establishing production facilities. This investment has contributed to a noticeable increase in the local supply of essential radiopharmaceuticals. It is noteworthy that several centers of excellence in NM are currently involved in providing early experience with innovative theranostic agents, despite several limitations. This has been made possible due to flexible national and institutional regulations that have facilitated the early adoption of many novel theranostic pairs. A notable example of this was the recent publication of the first-in-human single photon emission computed tomography /computed tomography of ^161^ terbium-prostate-specific membrane antigen (PSMA) in metastatic castration-resistant prostate cancer by the King Hussein Cancer Center team. 10 11

In many developing countries, NM specialists are often perceived by other specialties as diagnostic physicians. This perception remains prominent in regions where the infrastructure for training, education, and fellowship programs in theranostic practice is insufficient. Consequently, it is imperative to impart extensive guidance on the significance of theranostic practice through education, training, and accreditation. 12 To be eligible for theranostic applications, physicians who have been board-certified must undergo specialized training and accumulate significant practical experience while conforming to the specific curricula of their respective countries to attain authorization as theranosticians. Several advanced countries are currently implementing licensing programs to facilitate the transition of NM practitioners into theranostic practice. Developing countries such as the Philippines and Jordan are also adopting this same approach by creating fellowship and accreditation programs. The International Atomic Energy Agency (IAEA) held a consultation meeting to establish a consistent curriculum for theranostics programs that can be implemented in both developed and developing countries. The sharing of research and knowledge in multicentric settings may be a viable method of bridging gaps and achieving uniformity in the provision of theranostic services. This was found to be the primary cornerstone that provided the initial spark for the early practice of lutetium PSMA in many countries. 13 Furthermore, the implementation of continuous professional development initiatives aimed at promoting interdisciplinary collaboration among medical specialties has demonstrated great potential. Such initiatives can cultivate knowledge surrounding theranostic techniques for appropriate patients, ultimately resulting in a heightened rate of referrals to NM departments.

In the field of theranostics, radiochemists play a crucial role, as they are responsible for producing radio-diagnostic agents for clinical application and overseeing in vitro and radioimmunoassay laboratories. Regrettably, there is a shortage of skilled radiochemists and radiopharmacists, a problem that is not only confined to developing countries but is also widespread in developed ones. 14 The shortage of trained personnel in radiopharmacy necessitates the creation of innovative educational programs that can help bridge the gap between basic research and clinical investigation. Moreover, there is a need for support to develop radiopharmacists who possess expertise in clinical research and regulatory pathways that pertain to investigational new theranostic applications and approvals. These matters are crucial for the advancement and prosperity of the field.

Countries that have implemented the theranostics approach at an early stage and have developed their ability to conduct in-house radiolabeling and quality control assurance may be able to establish self-dependency under local regulatory frameworks and international ethical and radiation safety standards. This can facilitate the introduction of new radiopharmaceuticals for patients to benefit from novel treatments that are not yet widely available elsewhere, thereby promoting the application of theranostics on a global scale. 11 Additionally, investing in research nuclear reactors that can produce radioisotopes used in theranostics shows great potential for developing countries like Jordan. 15

The recognition and implementation of theranostic practice can differ greatly based on the availability of NM imaging methods and regulatory processes. In developing countries, centers that lack adequate resources may face fewer difficulties in meeting regulatory requirements and accreditation due to the more lenient regulatory practices in these countries. Nonetheless, the mere establishment of NM facilities cannot effectively address the issue at hand. Newly established centers must ensure adequate equipment and staff and be strategically located in densely populated urban areas to meet demand. The failure of conventional medical oncologists to promote theranostic practice may be attributed to their lack of exposure to nuclear physicians who specialize in therapy, resulting in a lack of knowledge and understanding. This inaccessibility may be due, in part, to the failure of NM theranosticians to attend tumor boards or multidisciplinary clinical meetings. Lack of national collaboration between medical specialties and the unavailability of the local NM community can lead to confusion in managing cases requiring theranostics, potentially limiting access to theranostic agents. As a result, many of these patients will end up getting alternative and sometimes suboptimal therapy without knowing about the value or availability of theranostics. Additionally, insurance coverage limitations may prevent patients from accessing theranostic care in some developing countries. Another important aspect of ensuring the continuous and successful development and distribution of novel theranostic agents is reimbursement. It is crucial to ensure that adequate reimbursement is provided not only for the agent itself but also for the associated medical and technical procedures. Failure to establish sufficient reimbursement has resulted in clinically useful drugs potentially failing in the past.

In comparison to traditional chemotherapies, novel theranostic agents offer a more targeted approach and hold the promise of reducing adverse side effects. Although the physical toxicity profile of these agents may have improved, their rising costs have become a significant concern. The incorporation of theranostic agents in nuclear oncology has faced challenges due to their association with the increasing expenses of cancer therapies. Additionally, the current regulatory environment has hindered advancements, resulting in an unfavorable and unsustainable situation. Additional expenses usually encompass licensing for radioactive materials, the implementation of necessary infrastructure, and dedicated spaces for isolation rooms. Concurrently, training and deployment of staff are needed to ensure the safe and efficient delivery of theranostics and the proper disposal of radioactive waste. The acquisition of imaging equipment and the establishment of an accredited radiation safety team also incur additional costs that need to be taken into account. This poses a major disadvantage in terms of the availability of many novel agents in certain developing countries with financial difficulties or conflicts. Overcoming these financial challenges is crucial to enhancing the feasibility of theranostics and ensuring their availability and accessibility to a broader range of cancer patients.

The fact that many developing countries rely on importing radiopharmaceuticals from distant countries also poses another logistics challenge, which can lead to nonadherence to therapy protocols and the pursuit of alternative approaches. The availability of imported radiopharmaceuticals is largely determined by the balance between supply and demand. This can sometimes complicate the availability of many recent radiopharmaceuticals. Despite growing interest in using ^223^ radium for bone palliation, it remains unavailable in many developing countries due to these challenges.

The previously mentioned challenges and barriers are exacerbated by a significant increase in the burden of cancer and other NCDs in developing countries. To address this issue, it is imperative to establish national and international professional cooperation, and international professional organizations such as the IAEA, the World Association of Radiopharmaceutical and Molecular Therapy, the International Conference on Radiopharmaceutical Therapy, and the International Center for Precision Oncology are expected to give a thrust to this advanced NM training and multidisciplinary practice in developing countries, which can provide an initial roadmap for reviving theranostic services in developing countries. The World Federation of Nuclear Medicine and Biology (WFNMB) is an important organization that plays a crucial role in advancing NM and theranostics worldwide. Its main goals are to promote high standards and best practices in NM while also advocating on behalf of the NM community with the Word Health Organization (WHO). WFNMB is the only nonstate NM organization that is recognized by the WHO, and it represents the interests of the NM community in various WHO forums. Through its collaboration with the WHO, WFNMB ensures that NM receives the attention it deserves as an essential medical specialty. The organization also raises awareness among policymakers, health care professionals, and the public about the potential benefits of NM in diagnosing and treating diseases. Through advocacy efforts, WFNMB aims to enhance patient care and outcomes worldwide by integrating NM into national health care systems, which is achieved through advocacy efforts. In addition to advocacy work, WFNMB promotes collaboration and knowledge exchange among professionals in NM and biology. This is done through facilitating networking opportunities, organizing international conferences, and promoting scientific research. WFNMB fosters interdisciplinary collaboration by connecting professionals from diverse backgrounds and regions to support the development of new techniques, technologies, and therapies in NM and related fields. Hence, it appears highly crucial to establish a collaborative network between developed nations and those with limited resources, as this can assist in bridging disparities and expediting a swift shift toward effectiveness and, ideally, self-sufficiency for numerous countries in the coming decades.

In brief, the employment of theranostic agents has gained popularity as a means to address the recent escalation of NCDs and curb their growth. However, developing countries continue to encounter a multitude of obstacles that demand immediate intervention. The inadequacy of resources, encompassing financial support, infrastructure, and skilled medical personnel, impedes the efficient administration of theranostic agents. Moreover, the lack of cooperation at a national level and inadequate availability of theranostic agents complicate the situation further. Barriers to patient access and the effective advancement and dissemination of innovative theranostic agents are posed by limitations in insurance coverage and reimbursement issues. These challenges can be overcome by fostering international collaboration and establishing a collaborative network. Therefore, it is essential for stakeholders, including professional organizations and policymakers, to collaborate in addressing these issues and ensuring fair access to theranostic care for individuals, regardless of their geographical location or socioeconomic status.
